# Immunomodulation after ischemic stroke: potential mechanisms and implications for therapy

**DOI:** 10.1186/s13054-016-1573-1

**Published:** 2016-12-07

**Authors:** Cynthia Santos Samary, Paolo Pelosi, Pedro Leme Silva, Patricia Rieken Macedo Rocco

**Affiliations:** 1 Laboratory of Pulmonary Investigation, Carlos Chagas Filho Biophysics Institute, Federal University of Rio de Janeiro, Centro de Ciências da Saúde, Avenida Carlos Chagas Filho, s/n, Bloco G-014, Ilha do Fundão, 21941-902 Rio de Janeiro, RJ Brazil; 2 Department of Surgical Sciences and Integrated Diagnostics, IRCCS AOU San Martino-IST, University of Genoa, Genoa, Italy

**Keywords:** Ischemic stroke, Immunosuppression, Stroke-associated pneumonia, Inflammation, Damage-associated molecular patterns

## Abstract

Brain injuries are often associated with intensive care admissions, and carry high morbidity and mortality rates. Ischemic stroke is one of the most frequent causes of injury to the central nervous system. It is now increasingly clear that human stroke causes multi-organ systemic disease. Brain inflammation may lead to opposing local and systemic effects. Suppression of systemic immunity by the nervous system could protect the brain from additional inflammatory damage; however, it may increase the susceptibility to infection. Pneumonia and urinary tract infection are the most common complications occurring in patients after stroke. The mechanisms involved in lung-brain interactions are still unknown, but some studies have suggested that inhibition of the cholinergic anti-inflammatory pathway and release of glucocorticoids, catecholamines, and damage-associated molecular patterns (DAMPs) are among the pathophysiological mechanisms involved in communication from the ischemic brain to the lungs after stroke. This review describes the modifications in local and systemic immunity that occur after stroke, outlines mechanisms of stroke-induced immunosuppression and their role in pneumonia, and highlights potential therapeutic targets to reduce post-stroke complications. Despite significant advances towards a better understanding of the pathophysiology of ischemic stroke-induced immunosuppression and stroke-associated pneumonia (SAP) in recent years, many unanswered questions remain. The true incidence and outcomes of SAP, especially in intensive care unit settings, have yet to be determined, as has the full extent of stroke-induced immunosuppression and its clinical implications.

## Background

Stroke is the second leading cause of death worldwide, and will affect at least one sixth of all persons at least once in their lives [1]. Patients with ischemic stroke require intensive care unit (ICU) admission for constant monitoring. While mortality rates have decreased recently, the incidence is increasing, and is predicted to reach 23 million cases by 2030 [2]. Furthermore, stroke is the leading cause of adult disability, with approximately one third of patients who survive 6 months becoming dependent on others [3]. Approximately 80% of all strokes are ischemic, resulting from thromboembolic occlusion of a major cerebral artery (usually the middle cerebral artery) or its branches.

Stroke deprives cells of their nutrient supply, causing immediate cell death and brain damage; the inflammatory process that follows exacerbates neurological deficits. Besides local inflammatory immune responses in the brain, stroke also alters systemic immunity, predisposing patients to immunosuppression and infections (mainly pneumonia and urinary tract infections), which are associated with poorer functional outcomes and increased morbidity [4]. However, whether these infections can be attributed to post-stroke immunosuppression or to other mechanisms remains unclear. Autoimmunity to central nervous system (CNS) antigens, overactivation of beta-adrenergic stimulation, and the cholinergic anti-inflammatory pathway are implicated in the immune dysfunction that follows acute stroke [5]. A greater understanding of the pathophysiological processes observed in the brain and periphery, during and following a stroke, is urgently needed to support development of potential therapies that could improve outcomes. This review describes the changes in local and systemic immunity that occur after stroke, outlines mechanisms of stroke-induced immunosuppression (with particular emphasis on its role in pneumonia), and highlights potential therapeutic targets for reduction of post-stroke complications.

## Pathophysiology of stroke

**Fig. 1:**
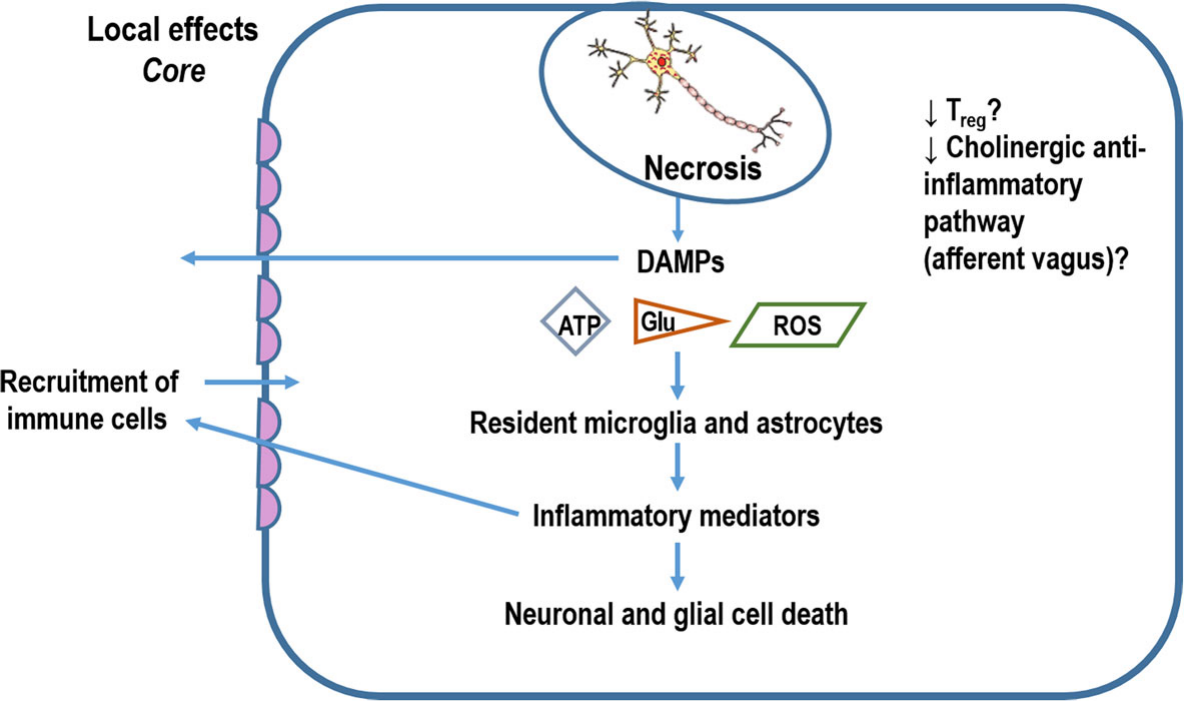
In the ischemic core, energy failure induces local excitotoxicity and peri-infarct depolarization, which lead to cell death, mainly by necrosis. The necrotic cells release several endogenous damage-associated molecular patterns (*DAMPs*): glutamate (*Glu*), reactive oxygen species (*ROS*), and adenosine triphosphate (*ATP*), which activate resident microglial cells and astrocytes to trigger downstream inflammatory signaling cascades. These mediators trigger the recruitment of peripheral immune cells in an attempt to initiate clearance of cell debris and healing in the brain. This process will induce further neuronal and glial cell death

**Fig. 2:**
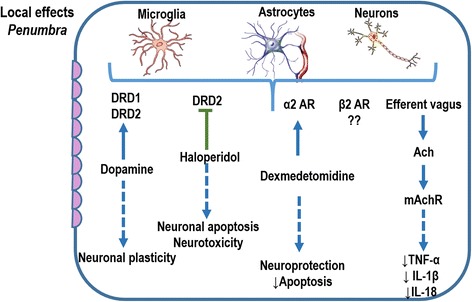
In the ischemic penumbra, excitotoxicity and peri-infarct depolarization lead to cell death by necrosis or apoptosis. Microglia, astrocytes, and neurons express receptors which can be activated or inhibited by some therapies, thus resulting in neuroprotection in this area of penumbra. *Solid blue lines* represent stimulation of the receptors. *Dashed blue lines* represent the effects resulting from stimulation. *Solid green lines* represent inhibition of the receptor. Dopamine receptor D1 (*DRD1*), dopamine receptor D2 (*DRD2*), α2 adrenergic receptor (*ARα2*), β2 adrenergic receptor (*ARβ2*), acetylcholine (*Ach*), muscarinic acetylcholine receptor (*mAchR*), tumor necrosis factor (*TNF*)-α, interleukin (*IL*)-1β, and IL-18

Ischemic stroke is characterized by a reduction in cerebral blood flow (CBF) that leads to metabolic and functional deficits. The ischemia itself is a result of occlusion of an artery supplying a specific brain territory. The infarct core is the central region of the brain to which blood flow is lost. The area surrounding this core, known as the ischemic penumbra, retains residual perfusion from collateral blood vessels. The penumbra retains structural integrity, but it is nonfunctional. Processes of cellular injury and death in these two regions are different [6]. Necrosis and apoptosis, two major modes of cell death, are implicated in ischemia. Necrosis is predominant in core tissue, whereas both necrosis and apoptosis are dominant in the penumbra [6] (Figs. 1 and 2).

Immediately after vascular occlusion, a complex pathophysiological process occurs, which may last hours or even days. CBF occlusion causes oxygen and glucose deprivation, resulting in neuronal death at the ischemic core via excitotoxicity [7]. Moreover, cerebral ischemia causes the release of an active neuropeptide (glutamate) that acts on N-methyl-D-aspartate (NMDA) receptors (NMDARs), thus causing an influx of calcium, which leads to formation of free radicals and induction of protein endonucleases. Excessive stimulation of NMDARs during ischemia contributes to mitochondrial injury, apoptotic, and excitotoxic nerve cell death [8]. Additionally, ischemia impairs ATP synthesis, thus preventing glutamate clearance, leading to continuous stimulation of glutamate receptors while keeping neuronal depolarization constant, generating reactive oxygen species (ROS) and mitochondrial dysfunction, stimulating necrotic and apoptotic pathways [6].

Dying cells release damage-associated molecular patterns (DAMPs) that stimulate the pathogen recognition receptors toll-like receptor (TLR)-2 and TLR-4, activate NF-κB and mitogen-activated protein (MAP) kinase pathways, and, consequently, produce pro-inflammatory mediators - interleukin (IL)-1β, IL-6, tumor necrosis factor (TNF)-α, chemokines - and their receptors [9]. These mediators trigger recruitment of peripheral immune cells in order to clear cell debris and heal the brain.

### The role of macrophages, neutrophils, and lymphocytes after stroke

Stroke disrupts the blood–brain barrier (BBB), increasing its permeability and the entry of immune cells [10]. Within 24 h after stroke onset, macrophages enter the brain, but their role in infarct development is controversial. Some studies have shown that macrophages exert neuroprotective effects through the production of transforming growth factor (TGF)-β, and stimulate healing and debris clearance [11]. Nevertheless, other studies suggest that macrophages can be neurodegenerative [11], because they are also a major source of IL-1β, TNF-α and ROS [12]. The controversial role of macrophages in infarct development may be explained by the concept of classic or alternative macrophage activation, whereby classically activated (M1) macrophages exacerbate damage, while alternatively activated (M2) macrophages assist in repair and neurogenesis [11].

The role of neutrophils in brain damage following stroke is equally controversial. Preventing neutrophil migration into cerebral ischemic tissue has been shown to reduce infarct size and improve neurological outcomes in mice [13]; however, neutrophil depletion has not been found to reduce brain damage after stroke [14]. While N1 neutrophils are present in the brain of mice post stroke, therapeutic activation of peroxisome proliferator-activated receptor (PPAR)γ has been shown to polarize neutrophils toward the N2 phenotype, resulting in neutrophil clearance and resolution of inflammation, thus promoting neuroprotection [15].

Influx of lymphocytes usually occurs 2–3 days after stroke. While T-cells produce IL-1β, interferon (IFN)-γ, and macrophage inflammatory protein (MIP)-2, thus contributing to infarct development, B cells may be neuroprotective, likely due to increased IL-10 [16]. Regulatory T-cells (T_regs_) also have controversial effects in stroke. T_regs_ can be strong promoters of ischemia or may lead to protective effects, mitigating infarct development. In this context, T_reg_ depletion has been shown to result in exacerbation of brain damage, probably due to reduced IL-10 production and increased lymphocyte function-associated antigen 1 (LFA-1)/intercellular adhesion molecule-1 (ICAM-1) [17].

## The specific immune response to ischemic stroke

The local and systemic immune response to ischemic stroke appears to differ from that mounted after injury to other organs or systems. Although the precise reasons remain unclear, several hypotheses could explain these putative differences.

During homeostasis, the brain is considered an immune-privileged tissue protected by a specific vessel structure, the BBB. During ischemic stroke, the BBB is breached and various immune cells (including both resident and peripheral cells) are recruited to the affected area. Ischemic stroke is also thought to influence immune cells in the circulation, possibly through increased activation of the sympathetic nervous system and the hypothalamic–pituitary–adrenal (HPA) axis. This may lead to a reduction in circulating immune cell counts and increase the risk of infectious complications [18].

During ischemic stroke, self-epitopes protected by the systemic immune system through different mechanisms may become open to adaptive immunity. This, in turn, may modulate the immune system to respond to self-antigens in the central nervous system (CNS), thus leading to autoimmunity. Therefore, stroke-induced immunosuppression helps prevent post-injury autoimmunity against CNS antigens [19]. Further studies are required to better elucidate the timing of inflammatory and anti-inflammatory responses in the brain.

Ischemic stroke without systemic inflammation may lead to active depression of peripheral immunity by the brain. In the absence of systemic inflammation but in the presence of local inflammatory cytokines after brain injury, an anti-inflammatory response may be triggered that is detrimental, because it shuts down defense mechanisms, rendering the body susceptible to infection. Under these conditions, the response could in fact be considered maladaptive or inefficient [4, 20].

Finally, unlike brain damage, other types of organ injury induce a potent stimulus of anti-inflammatory signaling by the CNS. Pro-inflammatory cytokines released during inflammation play a central role in wound healing. To control inflammation, the CNS sets up a homeostatic, counter-regulatory anti-inflammatory response, and the brain–immune interaction helps maintain homeostasis [21].

## Systemic immunosuppression

As noted above, stroke is associated with immune consequences, including local autoimmunity and peripheral immunosuppression. Systemic inflammation, characterized by increased IL-6 and IFN-γ production, may peak within 6 h of stroke, but systemic immunosuppression occurs so as to compensate for brain damage [22].

Local autoimmunity contributes to lesion formation. In experimental stroke, inhibition of this response has been shown to be beneficial. However, severe peripheral immunosuppression predisposes patients to subsequent bacterial infections, which have a negative effect on clinical outcome. The immune and molecular mechanisms responsible for this systemic immune activation have yet to be elucidated.

### Autoimmunity

Post-stroke immunosuppression has been considered an adaptive response, which prevents autoimmunity against CNS antigens. However, the mechanisms associated with stroke-related autoimmunity to brain antigens require elucidation. Reaction to stroke injury appears to involve a maladaptive response. During stroke, self-epitopes, protected by the systemic immune system through different mechanisms [23], could become open to adaptive immunity, which may modulate the immune system to respond to self-antigens in CNS, thus leading to autoimmunity. Therefore, stroke-induced immunosuppression helps prevent post-injury autoimmunity.

Post-stroke infections result in systemic inflammation, making patients more prone to autoimmunity against brain antigens [20]. However, immune responses against self-antigens may happen as a collateral effect. Post-stroke infection after endotoxin administration in rats increases both T helper (Th)1-mediated immune response and mortality rates [24]. Furthermore, stroke patients with acquired infection appeared to present immune responses against myelin basic protein and glial fibrillary acidic protein, and have worse outcomes compared to stroke patients with no infection [25]. This may emphasize the need to decrease the incidence of post-stroke infection.

### Cellular mechanisms involved in systemic immunosuppression

Central nervous system damage affects the immune response, as demonstrated in humans after neurosurgery, acute traumatic brain injury, and spinal cord injury [21]. In an experimental mouse model, it was demonstrated that stroke induces rapid and long-lasting suppression of cellular immune responses [18]. In humans, alterations in cell-mediated immunity, including decreased peripheral blood lymphocyte counts and impaired mitogenic T cell responses, have been reported after acute ischemic stroke [26]. In ischemic stroke, impairment of respiratory burst caused by circulating neutrophils and monocytes has also been observed. Additionally, stroke-induced neutrophil alterations are associated with the catecholamine response [27].

By linking the innate and adaptive immunity, macrophages interact with T-cells to trigger specific immune responses. After stroke, a reduction in the expression of major histocompatibility complexes (MHC) class II (or HLA-DR in humans) and in co-stimulation efficacy occurs, with production of pro-inflammatory cytokines [28]. In short, stroke affects immune system function, impairing bactericidal immunity and thus predisposing the host to bacterial infection.

Moreover, immune organ size is reduced after stroke, which suggests immunosuppression [18]. In this line, stroke has been shown to decrease spleen size due to splenocyte death, which decreases production of T cell mitogenic factors, preventing T cell proliferation and production of pro-inflammatory cytokines [18], and migration of cells from the spleen to the cerebral parenchyma [29].

In the post-stroke phase, a T cell shift from a cell-mediated inflammatory Th1-type response to a humoral mediated anti-inflammatory Th2-type response occurs, probably to protect the brain from additional inflammatory injury, and to stimulate tissue repair and neuronal regeneration [30]. On the other hand, this shift results in immunosuppression of the host, resulting in increased post-stroke infection susceptibility.

Experimental and clinical studies show that monocytes, dendritic cells, and T_regs_ greatly increase secretion of IL-10 after stroke [31, 32], averting a pro-inflammatory response. Additionally, IL-10 has been shown to inhibit production of IFN-γ by Th1 cells, of TNF-α by macrophages, and of cytokines by monocytes, and proliferation of T cells and cytokine responses, which emphasizes the role of IL-10 function in the immune shift toward a Th2 response and immunosuppression after stroke [33].

Based on the foregoing, innate immune cells become a desirable target for the treatment of stroke. Clarifying the complex crosstalk between immune responses and the nervous system is important to developing new therapies capable of reducing inflammation and improving recovery from stroke.

### Sympathetic nervous system

**Fig. 3:**
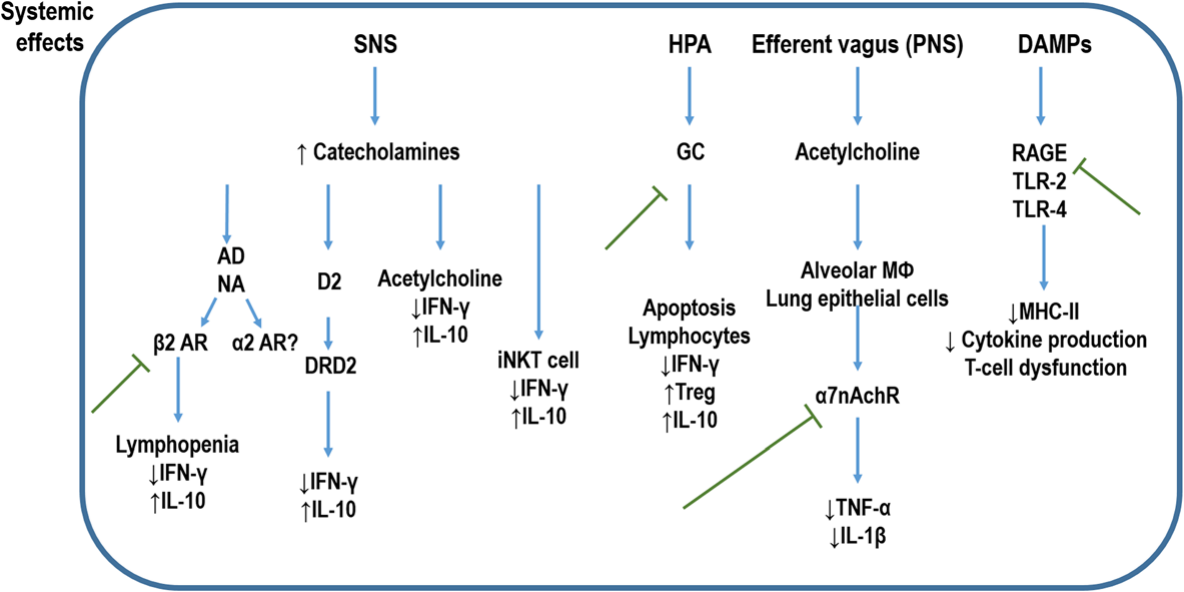
Ischemic stroke induces systemic immunosuppression mediated by several factors. These are: (1) overactivation of the sympathetic nervous system (*SNS*) results in secretion of catecholamines, which act on β-adrenergic receptors (*ARβ2*), dopaminergic receptors (dopamine receptor D2, *DRD2*), acetylcholine, and hepatic invariant natural killer T-cell (*iNKT*) stimulation; (2) activation of the hypothalamic-pituitary-adrenal (*HPA*) axis results in excessive glucocorticoid (*GC*) secretion, which acts on T cells to reduce interferon (*IFN*)-γ production, inducing apoptotic cell death and promoting interleukin (*IL*)-10 secretion through regulatory T cells (*T*_*reg*_); (3) parasympathetic nervous system (*PNS*) stimulation activates the cholinergic anti-inflammatory pathway, driven by the efferent vagus nerve at nAChRα7 receptors expressed in alveolar macrophages (*Mθ*) and lung epithelial cells, reducing tumor necrosis factor (*TNF*)-α and interleukin (IL)-1β; and (4) damage-associated molecular patterns (*DAMPs*) are released by cells undergoing non-apoptotic death or by immune system cells, characterized by reduced major histocompatibility complex (*MHC*)-II expression, decreased cytokine production upon stimulation, and T cell dysfunction. This contributes to the overall immunosuppressive state after stroke, which is the main explanation for post-stroke susceptibility to infection. *Blue arrows* represent the systemic effects of stroke. *Green lines* represent possible therapeutic targets to prevent systemic immunosuppression and stroke-associated pneumonia. *AD* adrenalin, *NR* noradrenaline, *RAGE* receptor for advanced glycation end-products, *TLR* toll-like receptor

The sympathetic nervous system (SNS) has a critical role in the communication between neural and immune structures (Fig. 3). Overactivation of the adrenergic nerve terminals is regarded as an advanced post-stroke immunosuppression mechanism, which induces activation of the SNS and leads to catecholamine secretion by the adrenal medulla and nerve terminals in peripheral organs [34]. Although increased catecholamine blood levels have been reported after experimental and clinical stroke [4, 35], their correlation with lymphopenia, humoral immunosuppression, or bacterial infection has not been confirmed in stroke patients [36]. Catecholamines act through β-adrenergic receptors on immune cells to decrease TNF-α and increase IL-10. The use of β-adrenergic receptor antagonists (β-blockers) in post-stroke mice led to reduced bacterial complications and mortality rates, which may suggest the importance of catecholamines in immunosuppression after stroke [18]. Moreover, hepatic invariant natural killer (iNKT) cells are the primary detectors and responders to distal brain damage [34], and their behavior is affected by post-stroke β-adrenergic signaling. β-adrenergic receptor blockade has also been shown to increase production of IFN-γ and decrease bacterial burden [34].

Dopamine is another catecholamine that can be released after stroke. Stimulation of D1 and D2 receptors results in immunomodulation via downregulation of NF-κB expression [37]. On the other hand, there are studies reporting that stimulation of these receptors activates NF-κB in a time-dependent and dose-dependent manner [38]. Even though the immunomodulatory effects of noradrenaline and adrenaline are well-established, the influence of dopamine on inflammatory responses remains unclear. However, dopamine might be a promising target in stroke therapy.

Alpha-adrenergic receptors are also expressed by most inflammatory cells and located in the CNS. Stimulation of α1-adrenoreceptors does not appear to have any effect on inflammatory responses. However, activation of α2 receptors markedly affects inflammatory cells, stimulating the release of a variety of pro-inflammatory and anti-inflammatory cytokines [39]. Conversely, α2 agonists have been associated with dose-dependent protection against brain matter loss in vivo and appear to improve the neurologic functional deficit induced by hypoxic-ischemic insult via α2 activation [40]. Thus, after stroke, modulation of sympathetic activity may influence brain and lung damage differentially. These complex interactions still need to be clarified.

### Hypothalamic–pituitary–adrenal axis

Briefly, the HPA axis is a set of CNS and endocrine structures that co-regulates bodily functions through the release of corticotropin-releasing hormone (CRH) by the hypothalamus in response to stress [41]. Additionally, cytokines (IL-1β, TNF-α, and IL-6) secreted by brain cells can stimulate the synthesis of CRH through specialized neurons [42]. Stroke induces production of these pro-inflammatory cytokines, which in turn can be sensed by the hypothalamus, resulting in excessive glucocorticoid secretion [43]. Glucocorticoids may affect T cells (reducing production of IFN-γ and inducing apoptosis) and monocytes (promoting secretion of IL-10). These effects may explain the lymphocyte apoptosis and lymphopenia observed after stroke [18]. Both low and high levels of cortisol are clinically associated with higher mortality after stroke [44], and the role of this hormone in the modulation of post-stroke inflammation is still unclear.

In summary, the marked anti-inflammatory responses that occur with sympathetic system and HPA axis activation result in strong glucocorticoid secretion. This dysregulation of the corticosteroid system induces immunosuppression, and might be associated with secondary infections and poor outcomes after stroke.

### Parasympathetic nervous system

The main function of the vagus nerve is to regulate the parasympathetic nervous system (PNS), which is also involved in the control of inflammation [45]. Vagus nerve stimulation (VNS) has been shown to modulate cerebral and systemic inflammation by release of noradrenaline (NA) and activation of the cholinergic anti-inflammatory pathway [46], which is driven by the action of the efferent vagus nerve at nicotinic acetylcholine receptor α7 (nAChRα7). After stroke, nAChRα7 stimulation reduces inflammation (regulating microglial activation in the brain), protects neuronal cells from oxidative stress, and improves functional recovery [47]. Furthermore, in the post-stroke phase, melanocortins regulate the cholinergic anti-inflammatory pathway and downregulate TNF-α [48].

It is known that resident lung immune cells, such as alveolar macrophages and alveolar epithelial cells, express α7nAChR [49]. This peripheral immune stimulation can reduce inflammation in the lungs [50], but can also causes sickness behavior, acting as a negative feedback loop to prevent a potentially harmful overreaction of the immune system during inflammatory conditions (such as bacterial infections) by suppressing production of pro-inflammatory cytokines by activated macrophages, thus impairing host defense [51].

### Danger-associated molecular patterns

After stroke, neuronal, glial, and vascular compartments are damaged, and this can trigger inflammasome and innate immune activation through the release of DAMPs [34]. Alarmins (high-mobility group box 1 (HMGB1), heat shock proteins, adenosine triphosphate (ATP), S100B proteins, heparin sulfate, DNA, RNA, oxidized low-density lipoprotein β-amyloid, and hyaluronan) are endogenous DAMPs released by cells undergoing non-apoptotic death (or by immune system cells), which trigger a sterile immune reaction designed to restore tissue homeostasis. After stroke, cells in the hypoperfused territory become necrotic, release HMGB1, induce expansion of a monocyte subpopulation, and contribute to the immunosuppressive state in the subacute phase of stroke, predisposing patients to pneumonia [52]. A recent experimental study showed that genetic or pharmacological blockade of pattern recognition receptor signaling via HMGB1 and receptor for advanced glycation end products (RAGE) abrogated cellular immunosuppression and restored lymphocyte activation in the subacute phase after stroke [53].

Furthermore, TLR-2, TLR-4, and RAGE are expressed by many cell types, and have been shown to mediate the inflammatory effects of HMGB1 through activation of NF-κB. After stroke, damaged astrocytes release RAGE, leading either to neuronal survival or death (depending on the level of NF-κB transcriptional activity) [54].

In short, activation of the DAMP system after stroke induces a key mechanism of the intricate local and peripheral immune response that has profound consequences on clinical outcome. Future treatments for stroke can target inflammasome signaling through the NF-κB and MAPK pathways, targeting purinergic receptors, ion channels, cytokines, and cytokine receptors.

### Stroke-associated pneumonia

Pneumonia is the most common type of infection after stroke, with an incidence of up to 22%, and has been shown to worsen clinical and neurological outcomes [55]. Stroke-associated pneumonia (SAP) has been classified as acute (occurring within 1 month of stroke) or chronic (occurring 1 month after stroke or later) [56]. Given this high incidence, recent studies have sought to investigate the mechanisms involved in development of pneumonia after stroke.

Traditionally, SAP was considered secondary to the aspiration and dysphagia that result from impaired swallowing function and involved a variety of aspects, including decreased level of consciousness, body positioning in bed, mechanical ventilation, and patient immobility [57]. However, the high incidence of pneumonia in post-stroke patients when compared to that observed in patients with only dysphagia or impaired consciousness suggests that other immunological mechanisms are implicated in the pathogenesis of SAP [4, 58]. Many stroke patients do have impaired swallowing function, which can be related to inadequate dopamine transmission [58]. In this line, D1 dopamine receptor blockade has been shown to inhibit the swallow reflex and reduce substance P secretion by the glossopharyngeal nerve in distal organs [59]. Low sputum levels of substance P, which is responsible for the coughing and swallowing reflex, have been observed in elderly patients who developed pneumonia after aspiration [58]. Accordingly, an increase in serum levels of substance P after treatment with an angiotensin-converting enzyme inhibitor was associated with a reduction of aspiration, suggesting an additional role of lower substance P levels in broncho-aspiration [58]. It has been suggested that chronically (after 1 month), the mechanisms of post-stroke pneumonia are related to apparent aspiration and dysphagia-associated micro-aspiration [56].

Aspiration is not the only cause of SAP as it has been shown to occur in healthy individuals to a similar extent as that observed in stroke patients, though pneumonia does not develop [60]. The oral flora change rapidly after stroke and colonization by Gram-negative bacteria occurs more frequently than in non-stroke patients [61]. Different bacterial species have been isolated from the sputum of stroke patients, including *Streptococcus pneumoniae*, *Staphylococcus aureus*, *Klebsiella pneumoniae*, *Pseudomonas aeruginosa, Escherichia coli* and *Enterobacter cloacae* [62]. Even though *E. coli* has been found in more than 95% of blood and lung cultures obtained from mice post stroke [18], the causative agent of infection cannot be identified in a large portion of patients post stroke [52]. It is important to elucidate the mechanisms and identify the causative agents of SAP in order to develop specifically targeted therapies to mitigate post-stroke bacterial complications.

## Perspective therapies

Currently, the only validated therapy for ischemic stroke is thrombolysis, which must be administered within 4–5 h of symptom onset [63]. Due to its narrow therapeutic time window and concerns about hemorrhagic complications, thrombolysis is still not used regularly [64].

Stroke yields complex changes in the immune system through different mechanisms resulting in immunosuppression, which leaves the host susceptible to infections. Targeting these different pathways to prevent the immune shift that usually follows stroke may provide an avenue for adjunctive therapy to prevent and address post-stroke infections. In this context, experimental models of ischemic stroke have shown that stem cells can lead to decreased post-ischemic inflammatory damage and functional improvement [65]. Additionally, several clinical trials have demonstrated that stem cell therapy (including with mesenchymal stem cells, bone marrow mononuclear cells, and neural stem/progenitor cells) following stroke is feasible and safe, but the results have been controversial [66] due to variability in patient characteristics, timing of therapy, dose and type of cells delivered, and mode of treatment. Further studies at both bench and bedside are needed to understand the mechanisms underlying stem cell therapy, and improve its therapeutic efficacy in patients with stroke.

In experimental studies, therapies that block β-adrenergic receptors have been shown to reduce infections after stroke [18]. Similarly, post-stroke patients treated with β-blockers prior to ictus and during hospitalization were found to have a lower incidence of pneumonia and significantly reduced 30-day mortality than patients not receiving β-blockers [67]. Conversely, in a recent clinical trial, β-blocker treatment reduced ICU admission, but did not provide adequate protection against pneumonia, and was associated with greater 30-day mortality than no β-blocker use [68]. In short, further studies are required to clarify the effectiveness of β-blocker therapy for prevention of stroke-associated infections.

Cerebral ischemia causes release of glutamate and acts on NMDA receptors, which leads to cell death. Thus, it has been hypothetized that stopping this process at either glutamate release or NMDA receptor inhibition levels might reduce or minimize the adverse neuronal effects of ischemic stroke. However, The NMDAR is not always excitotoxic. This receptor is known to have dual effects; it promotes neuronal death or survival in primary neuronal cultures in vitro and in the rat brain in vivo, depending on the level of receptor activity [69]. This may explain why in some clinical trials using a low-affinity blocker of the NMDA ion channel, mortality in patients with acute ischemic stroke was not reduced [70].

Additional mechanistic and time-course studies on the brain–lung axis are needed to develop alternative therapeutic options. After stroke, suppression of the immune system seems to be a brain-protective mechanism, as the majority of penumbral damage is governed by inflammatory events. However, immunomodulatory treatments require caution, as they may prevent stroke-associated pneumonia but promote further collateral damage in the brain. An optimal immunomodulatory regimen should reduce infection burden without exacerbating brain damage.

## Conclusions

Despite significant advances towards a better understanding of the pathophysiology of ischemic stroke-induced immunosuppression and SAP in recent years, many unanswered questions remain. The true incidence and outcomes of SAP, especially in ICU settings, have yet to be determined, as does the full extent of stroke-induced immunosuppression and its clinical implications. Thus, future research should focus on elucidating the different pathophysiological mechanisms of stroke-related immunosuppression. Prospective clinical studies are needed to clarify the incidence of SAP, outcomes, and potential prophylactic strategies.

## Abbreviations

AEC: alveolar epithelial cells
ATP: adenosine triphosphate
BBB: blood–brain barrier
CBF: cerebral blood flow
CNS: central nervous system
CRH: corticotropin-releasing hormone
CTLA: cytotoxic T-lymphocyte-associated protein
DAMPs: danger-associated molecular patterns
HMGB1: high-mobility group box 1
HPA: hypothalamic–pituitary–adrenal
IFN-γ: interferon gamma
IL: interleukin
iNKT: invariant natural killer
LPS: lipopolysaccharide
MAP: mitogen-activated protein
MHC: major histocompatibility complexes
MIP-2: macrophage inflammatory protein-2
nAChRα7: nicotinic acetylcholine receptor alpha-7
NF-κB: nuclear factor kappa B
NMDAR: N-methyl-D-aspartate receptor
PNS: parasympathetic nervous system
RAGE: receptor for advanced glycation end products
ROS: reactive oxygen species
SAP: stroke-associated pneumonia
TGF: transforming growth factor
TLR: toll-like receptor
TNF: tumor necrosis factor
T_regs_: regulatory T cells
VNS: vagus nerve stimulation
