# Erratum to: Immunomodulation after ischemic stroke: potential mechanisms and implications for therapy

**DOI:** 10.1186/s13054-017-1834-7

**Published:** 2017-10-18

**Authors:** Cynthia Santos Samary, Paolo Pelosi, Pedro Leme Silva, Patricia Rieken Macedo Rocco

**Affiliations:** 1 Laboratory of Pulmonary Investigation, Carlos Chagas Filho Biophysics, Institute, Federal University of Rio de Janeiro, Centro de Ciências da Saúde, Avenida Carlos Chagas Filho, s/n, Bloco G-014, Ilha do Fundão, 21941-902 Rio de Janeiro, RJ Brazil; 2 Department of Surgical Sciences and Integrated Diagnostics, IRCCS AOU San Martino-IST, University of Genoa, Genoa, Italy

Following publication of this article [1] it came to our attention that we failed to reference a review article by Shim and Wong [2] which influenced our review for *Critical Care*. We sincerely apologize for this oversight.
